# Composition Tuning of Nanostructured Binary Copper Selenides through Rapid Chemical Synthesis and Their Thermoelectric Property Evaluation

**DOI:** 10.3390/nano10050854

**Published:** 2020-04-28

**Authors:** Bejan Hamawandi, Sedat Ballikaya, Mikael Råsander, Joseph Halim, Lorenzo Vinciguerra, Johanna Rosén, Mats Johnsson, Muhammet S. Toprak

**Affiliations:** 1 Department of Applied Physics, KTH Royal Institute of Technology, SE-106 91 Stockholm, Sweden; bejan@kth.se (B.H.); lorenzo.vinciguerra.25@gmail.com (L.V.); 2 Department of Physics, University of Istanbul, Fatih, Istanbul 34135, Turkey; 3 Applied Physics, Division of Materials Science, Department of Engineering Sciences and Mathematics, Luleå University of Technology, SE-971 87 Luleå, Sweden; mikael.rasander@ltu.se; 4 Department of Physics, Chemistry and Biology (IFM), SE-581 83 Linköping, Sweden; joseph.n.ernest@gmail.com (J.H.); johanna.rosen@liu.se (J.R.); 5 Department of Materials and Environmental Chemistry, Stockholm University, SE-106 91 Stockholm, Sweden; mats.johnsson@mmk.su.se

**Keywords:** thermoelectric, chalcogenides, Cu_2−x_Se, microwave synthesis, nanomaterial, XPS, ZT, thermal conductivity

## Abstract

Reduced energy consumption and environmentally friendly, abundant constituents are gaining more attention for the synthesis of energy materials. A rapid, highly scalable, and process-temperature-sensitive solution synthesis route is demonstrated for the fabrication of thermoelectric (TE) Cu_2−x_Se. The process relies on readily available precursors and microwave-assisted thermolysis, which is sensitive to reaction conditions; yielding Cu_1.8_Se at 200 °C and Cu_2_Se at 250 °C within 6–8 min reaction time. Transmission electron microscopy (TEM) revealed crystalline nature of as-made particles with irregular truncated morphology, which exhibit a high phase purity as identified by X-ray powder diffraction (XRPD) analysis. Temperature-dependent transport properties were characterized via electrical conductivity, Seebeck coefficient, and thermal diffusivity measurements. Subsequent to spark plasma sintering, pure Cu_1.8_Se exhibited highly compacted and oriented grains that were similar in size in comparison to Cu_2_Se, which led to its high electrical and low thermal conductivity, reaching a very high power-factor (24 µW/K^−2^cm^−1^). Density-of-states (DOS) calculations confirm the observed trends in electronic properties of the material, where Cu-deficient phase exhibits metallic character. The TE figure of merit (*ZT*) was estimated for the materials, demonstrating an unprecedentedly high *ZT* at 875 K of 2.1 for Cu_1.8_Se sample, followed by 1.9 for Cu_2_Se. Synthetic and processing methods presented in this work enable large-scale production of TE materials and components for niche applications.

## 1. Introduction

Thermoelectricity is the current flow due to a temperature difference across the material or vice versa. This concept is used to recover the waste heat generated by industrial- and smaller-scale applications and convert it directly to electricity. The reverse concept of generating thermal gradients, i.e., cold–hot sides, using the thermoelectric (TE) unit are indeed useful for many applications. TE systems in general are stable and reliable, with no maintenance requirements, due to having no moving parts. However, the performance limitation and material abundance are great motivations in searching for new TE materials and improving the existing ones. The efficiency of a TE material is typically represented by the dimensionless TE figure of merit, defined as *ZT* = (*S^2^σT)/κ*, where ***S***, ***σ***, and *κ* are the Seebeck coefficient, electrical conductivity, and total thermal conductivity, respectively, and *T* is the absolute temperature [1]. The record of *ZT* is continuously re-written in conventional systems like Bi_2_Te_3_ [2] and PbTe [3] that still dominate the industrial applications. *ZT* around 1 has served as a standard for niche applications; however, realistically, *ZT* > 2 is necessary for broad implementation of TE technology [4].

New, high-performance TE materials, especially with earth-abundant and low-toxicity constituents, are receiving significant attention, although they are not without their own challenges. Decent ***ZT*** values have been achieved in various compounds that are free from the scarce Te or the toxic Pb, such as half-Heusler compounds, skutterudites, Mg_2_X (X = Si, Ge, Sn), MgAgSb, BiCuSeO, SnSe, and Cu_2−x_Q (Q = Te, Se, S) [5,6,7,8,9,10,11,12]. Transition-metal chalcogenides are of current interest to energy-related research due to their semiconducting properties along with the ability to tune these properties through careful manipulation of the synthesis conditions. These materials have historically been made by using energy-intensive and ultra-high-vacuum solid-state techniques that result in a high fabrication cost. Cu_2−x_Se is a transition-metal chalcogenide which is found to be a promising material for medium-temperature-range TE applications. Cu/Se stoichiometry directly determines both phase composition and crystal structure. Cu_2−x_Se is a typical semiconductor with a band gap in the range of 1.2–2.3 eV (an indirect band gap of 1.1–1.5 eV and a direct band gap of 2.0–2.3 eV) [13,14]. The interest in this material is mainly due to its low thermal conductivity as well as its phase-changing nature [15]. The nanostructured bulk forms of Cu_2−x_Se have gained great attention recently due to their potential applications in waste-heat recovery [12,13,14,15,16,17,18,19,20].

Recent studies show that synthesis route may dramatically influence and contribute to TE properties of nanomaterials [21,22,23,24,25,26,27,28]. Among these methods, solvothermal, thermolysis [22,29,30,31,32] melting and annealing [24,26,33,34], mechanical alloying [35,36,37], co-precipitation and solution reduction [38], and microwave (MW)-assisted synthesis [14,32,39] have been reported in recent decades. MW-assisted synthesis has been reported for the synthesis of (BiSb)_2_(TeSe)_3_ systems, where the produced quantities per batch have been rather limited. In solution chemical synthesis, typically the free atomic or ionic species and their reaction allows the nucleation of particles. These processes are conventionally performed in anhydrous organic solvents with very high boiling points, reaching temperatures of 300–400 °C. A similar chemistry can be worked out using the MW-assisted heating at much more moderate temperatures due to different and effective volume heating or to the precursor activation mechanism with MW irradiation.

Synthesis schemes that are resource-effective and have reduced energy consumption are gaining much more significance due to the environmental impact of various synthesis processes. We recently reported a high-throughput MW-assisted synthesis method that yielded Cu_2_Se with a very promising *ZT* of 2 [32]. In the MW synthesis, the reaction conditions, especially the temperature, pressure, and MW power, can be fully controlled, monitored, and tailored to the desired synthesis procedures [21,32,35], significantly differing from autoclave-based processes. In this work, we aim at demonstrating the process sensitivity of MW-assisted synthesis exemplified by Cu_1.8_Se and Cu_2_Se phases for obtaining highly efficient nanostructured TE materials. The proposed method is rather fast, truly scalable, energy-efficient, and ideally controls the chemical route of the reaction, with a high degree of precision and reproducibility. It does not need standard Schlenk line and glovebox techniques that are used otherwise. By controlling the reaction temperature, nanosized Cu_1.8_Se and Cu_2_Se materials were synthesized at 200 and 250 °C, respectively. Detailed synthesis, processing, microstructural and transport property evaluation, and DOS calculations of the materials are presented, paving the way to high-efficiency nanostructured TE materials. 

## 2. Materials and Methods

### 2.1. Materials

All the chemicals were purchased from Sigma Aldrich (Stockholm, Sweden). Copper acetate (Cu(CO_2_CH_3_)_2_H_2_O, 98%), oleic acid (C_18_H_34_O_2_), 1-octadecene (C_18_H_36_, ODE), selenium powder (Se, 99.5%), and trioctylphosphine (P(C_8_H_17_)_3_, TOP) were used. Methanol and hexane (C_6_H_14_) were used for washing the synthesized particles. All chemicals were used as received, without further purification.

### 2.2. Synthesis and Consolidation of Cu_2−x_Se

**Figure 1:**
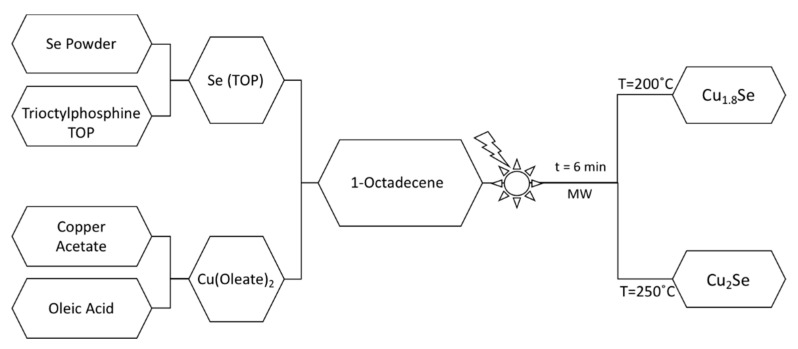
Schematic description of the microwave (MW)-assisted synthesis process of Cu_2−x_Se.

The synthesis scheme is briefly described in Figure 1 and is detailed in the Supplementary Materials. All the synthesis work was performed using MW-assisted heating process, which can be listed among the most energy-effective synthesis routes for the production of semiconductor nanoparticles, based on the thermal decomposition of chemical precursors that are otherwise stable at room temperature. In a typical process, the copper precursor (Cu-oleate) solution was prepared by dissolving a stoichiometric amount of copper acetate in oleic acid and octadecene under continuous stirring. A stoichiometric amount of Se powder was separately complexed with TOP until all the selenium was completely dissolved. The synthesis reactions were performed under MW-assisted heating (controlled temperature and pressure) at 200 and 250 °C, yielding Cu_1.8_Se and Cu_2_Se, respectively. The typical temperature and pressure profiles of the reaction are presented in Figure S1. The obtained powders were then washed several times using a mixture of methanol and hexane. Finally, the powders were placed in a vacuum oven to ensure total solvent evaporation.

The obtained powders were then compacted by spark plasma sintering (SPS, Dr Sinter 825, Fuji Electronic Industrial Co. Ltd., Japan) in a 15 mm diameter die to form pellets for TE transport property measurements. SPS conditions were previously studied by our group [32], and we adopted conditions similar to our earlier work. The powders were loaded in a graphite die for the spark plasma sintering (SPS) process, the parameters of which are listed in Table 1. A lower sintering temperature was chosen for Cu_1.8_Se than for Cu_2_Se, as the sintered pellets cracked upon sintering above 300 °C. Further details about pellets are presented in Table S1, which reveals that compaction density of >90% was achieved for both the samples.

### 2.3. Characterization Methods

#### 2.3.1. Transport Property Measurements

TE transport properties were determined via electrical conductivity (***σ***), Seebeck coefficient (***S***), and thermal diffusivity (*D*) measurements in the range of 300–875 K. The Seebeck coefficient ***S*** and the electrical conductivity *σ* were measured simultaneously on the pellets obtained after the SPS process, using a commercial instrument ZEM-ULVAC M8 model (ULVAC, MA, USA) which measures the *S* and ***σ*** based on the four-point-probe method. The total thermal conductivity (*κ*_tot_) was calculated using the following equation: $\kappa_{tot}$ = *D·ρ·C*_p_, where *D* is the thermal diffusivity, *C*_p_ is the specific heat capacity, and ***ρ*** is the bulk density (packing density, see Table 1) of the compacted pellet obtained from the Archimedean method. A laser flash analysis system (LFA 1000, Linseis Messgeraete GmbH, Germany) was used to measure the thermal diffusivity, ***D***, on a pellet of 13 mm diameter and 2 mm thickness. Heat capacity measurements were performed via differential scanning calorimetry (PT 1000, Linseis Messgeraete GmbH, Germany). The temperature range of transport measurement was from 300 to 875 K; with all the properties known, the TE figure of merit (*ZT*) was estimated.

#### 2.3.2. Structural and Morphological Characterization

X-Ray diffraction (XRD) (Panalytical Xpert Pro alpha powder, PANalytical BV, Almelo, Netherlands) was employed to identify the crystalline phases obtained both before and after compaction. The system utilizes a Cu K-alpha source at 1.5406 Å wavelength and scanning rate of 0.13°/min. The specific heat capacity of the sintered samples was measured by differential scanning calorimetry (DSC) (DSC404, Netzsch, Selb, Germany). Scanning electron microscopy (SEM) (FEI Nova 200, Hillsboro, OR, USA) was employed to analyze the morphology and particle size of the dried powders and the fracture surface of samples after compaction. Specimens were prepared on copper tape or graphene to avoid charging effects. Transmission electron microscopy (TEM) (JEM-2100F, 200 kV, JEOL Ltd. Tokyo Japan) was performed on the as-made nanopowders by dispersing 20 µL NP suspension on the Cu TEM grid and drying in air. X-ray photoelectron spectroscopy (XPS) measurements were performed on polished bulk pieces of Cu_2_Se and Cu_1.8_Se, using a surface analysis system (Kratos AXIS Ultra^DLD^, Manchester, U.K.) with monochromatic Al-Kα (1486.6 eV) radiation. Details of the XPS analyses are presented in the Supplementary Materials.

### 2.4. Band Structure Calculations

The calculations were performed using density functional theory as it has been implemented in the VASP code [40,41]. Standard PAW potentials [42] were used to describe both Cu and Se, and we used the PBE exchange correlation functional [43]. To account for the localization of the Cu 3d states, we used the “effective U” method of Dudarev et al. [44]. A plane wave energy cut-off of 800 eV was used for all calculations, and a 20 × 20 × 20 k-points mesh was used. For supercells (see below) the k-points mesh was adapted accordingly.

The high-temperature phase of Cu_2_Se was modeled in an anti-fluorite crystal structure with the $Fm\bar{3}m$ space group, where the Cu atoms occupies the tetrahedrally coordinated interstitial sites inside a face-centered cubic cage formed by the Se atoms. The lattice constant of Cu_2_Se with these settings is 5.718 Å, which is in reasonable agreement with previous calculations as well as with experimental data [45]. The deviation from the experimental lattice constant is about 1%, which is customary for this type of calculation [46].

To model Cu_1.8_Se, a 3 × 3 × 3 supercell of the primitive Cu_2_Se structure was used. The Cu_1.8_Se system was then modelled with the same structure as Cu_2_Se but with 10% of the Cu positions being vacant. The 3 × 3 × 3 supercell used has 54 Cu positions; in order to model 10% vacancies, five Cu atoms were removed from the lattice, thereby creating an effective vacancy concentration of 9%. We also tested a single Cu vacancy in Cu_2_Se for this supercell with essentially the same result as for the system with 9% vacancies. For the systems with vacancies, relaxations of the atomic positions were performed until the forces acting between the atoms were smaller than 10 meV/Å. Calculations for systems with Cu vacancies also allowed for a spin-polarized solution of the electronic structure.

## 3. Results and Discussion

In the synthetic process adopted here, Se is solubilized by complexing with TOP, forming Se(TOP), and Cu is complexed with oleic acid, forming Cu(Oleate)_2_. The reaction taking place during the formation of Cu_2−x_Se compounds under MW irradiation can be represented as:

2Cu(Oleate)_2_ + Se(TOP) → Cu_2−x_Se + (TOPO) + Oleic Anhydride + x Cu(Oleate)_2_; where x = 0, or 0.2(1)

At 200 °C, the Cu-deficient non-stoichiometric phase forms, where x is 0.2; at the slightly elevated temperature of 250 °C, the stoichiometric Cu_2_Se phase is obtained. One can see this reaction directly through the color change of the dispersion from a dark green-blue to a dark black-brown, with black-brown powder precipitates after the MW heating process. The process is schematically presented in Figure 1.

### 3.1. Structural Analysis

**Figure 2:**
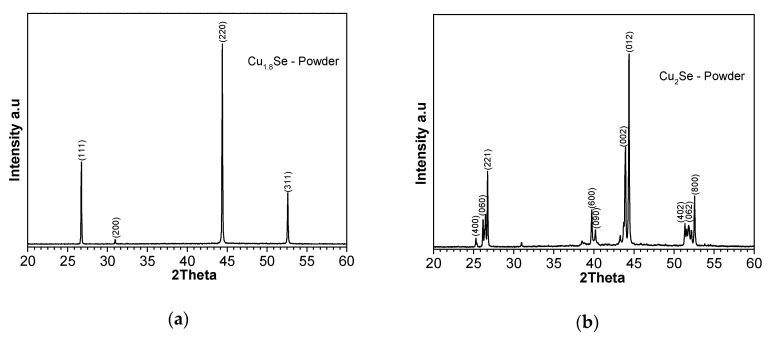
The room-temperature X-ray powder diffraction (XRPD) patterns of as-prepared powder and SPS sintered samples of (**a**) Cu_1.8_Se and (**b**) Cu_2_Se. Indexing was performed against Cu_2_Se phase with International Centre for Diffraction Data-ICDD card No. 27-1131, and Cu_1.8_Se phase with ICDD card No. 01-071-0044.

Phase purity of Cu_2−x_Se nanomaterials was investigated using X-ray powder diffraction (XRPD) analysis. Room temperature XRPD patterns for as-synthesized materials are presented in Figure 2. In general, the major crystal phases in both the bulk and in NCs are cubic berzelianite Cu_2_Se or Cu_1.8_Se for as-made materials. Indexing is performed against the target compositions of Cu_1.8_Se with cubic F_m-3m_ space group (International Centre for Diffraction Data -ICDD card No. 01-071-0044) and Cu_2_Se monoclinic crystal structure with space group P (ICDD card No. 27-1131). Major diffraction peaks are marked by their respective Miller indices for the pure phases. The materials showed a high phase purity, and no secondary phases were observed in the Cu_2_Se and Cu_1.8_Se compounds.

### 3.2. XPS Analyses

**Figure 3:**
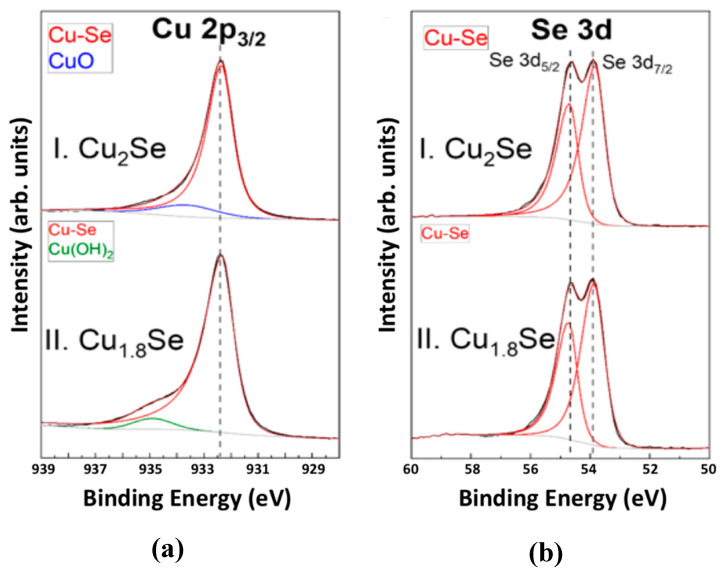
X-ray photoelectron spectroscopy (XPS) high-resolution spectra with peak fitting for (**a**) Cu 2p_3/2_ and (**b**) Se 3d regions for (I) Cu_2_Se and (II) Cu_1.8_Se bulk samples. Various peaks shown represent various species assumed to exist. Labels and peak colors are correlated. The global atomic percentages and the fitting results for Cu 2p_3/2_ and Se 3d regions are summarized in Table S1, Table 2, and Table S3, respectively.

XPS analyses were performed on SPS sintered pellets in order to establish the composition of compacted pellets for the Cu_1.8_Se and Cu_2_Se samples. High-resolution XPS spectra of Cu 2p_3/2_ and Se 3d regions—along with their peak fittings—of Cu_2_Se and Cu_1.8_Se bulk samples are plotted in Figure 3a,b. The peak fitting results for the various species are summarized in Table S2 and Table 2. The Cu 2p_3/2_ region for Cu_2_Se and Cu_1.8_Se bulk samples was fitted with two peaks, each corresponding to two species: Cu-Se bond and CuO surface oxide in the case of Cu_2_Se, with about 10% surface CuO. For Cu_1.8_Se, the XPS Cu 2p_3/2_ region was fitted by two peaks, corresponding to Cu-Se bond and Cu(OH)_2_ surface hydroxide, with about 5% Cu(OH)_2_. The binding energy (BE) of the Cu-Se species for the samples was exactly the same (932.3 eV), while the Se 3d region was fitted by a peak corresponding to the Cu-Se bond (Figure 3b). Binding energy (BE) of such species is close to that of elementary Se, indicating a covalent character of the Cu–Se bond in the Cu–Se compounds. BE of that species is shifted slightly to a higher value in case of increasing the relative Se content from Cu_2_Se to Cu_1.8_Se. These results show that the deviation from stoichiometry has no effect on the BE value of the Cu-Se species in the Cu 2p_3/2_ region and a slight effect on the BE value of the Cu–Se species in the Se 3d region (Table S3). These results are consistent with those presented in [4,37]. The minor phases of CuO and Cu(OH)_2_ are not expected to make a large impact on the observed transport properties, as they are more prone to be on the surface and are not identified by other analysis techniques. The atomic percentages of various species were determined by multiplying the total atomic percentage of each element by the fraction of that element. The total atomic percentage of each element (Table S1) was obtained from the survey spectra presented in Figure S2.

### 3.3. SEM and TEM Analyses

**Figure 4:**
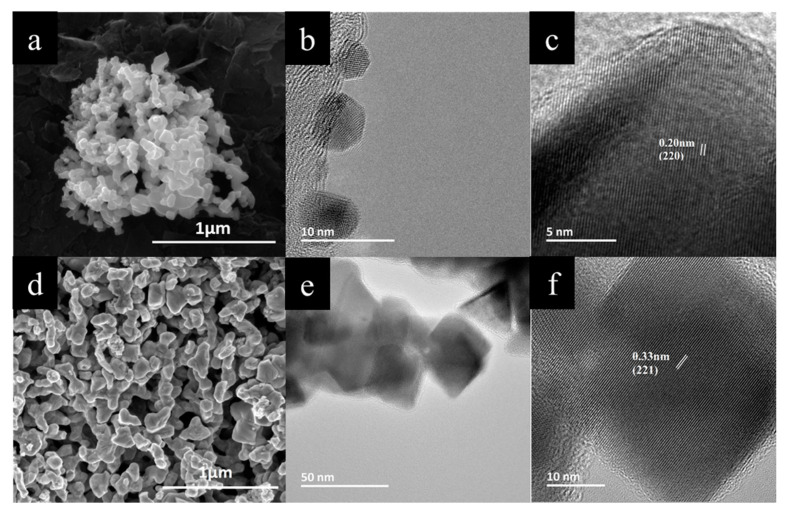
Scanning electron microscopy (SEM) micrographs of as-made Cu_1.8_Se (**a**) and Cu_2_Se (**d**); TEM micrographs of as-made Cu_1.8_Se (**b**–**c**) and Cu_2_Se (**e**–**f**) samples at different magnifications, revealing very fine particle size and high crystallinity. Lattice fringes in Figure 4c are measured as 0.2 nm and indexed for the (220) plane of α-Cu_1.8_Se (ICDD card No. 01-071-0044). Lattice fringes in Figure 4f are indexed for the (221) plane of the monoclinic α-Cu_2−x_Se (ICDD card No. 27-1131).

Microstructure of the samples has been investigated using SEM and TEM techniques. Figure 4a–f shows micrographs of as-made Cu_1.8_Se and Cu_2_Se samples. SEM micrographs reveal widely dispersed particle size ranging from <10 nm up to about 200 nm. A detailed investigation by TEM showed particle size of Cu_1.8_Se of around 4–6 nm. Lattice fringes are clearly visible in Figure 4c, measured as 0.2 nm and indexed for the (220) plane of α-Cu_1.8_Se structure (ICDD card No. 01-071-0044). Figure 4e,f reveals the particle size and internal structure for Cu_2_Se sample, where particle size of about 30 nm is observed, with a very high crystallinity. Lattice fringes in Figure 4f are indexed for the (221) plane of the monoclinic α-Cu_2_Se phase (matched with the ICDD card No. 27-1131).

**Figure 5:**
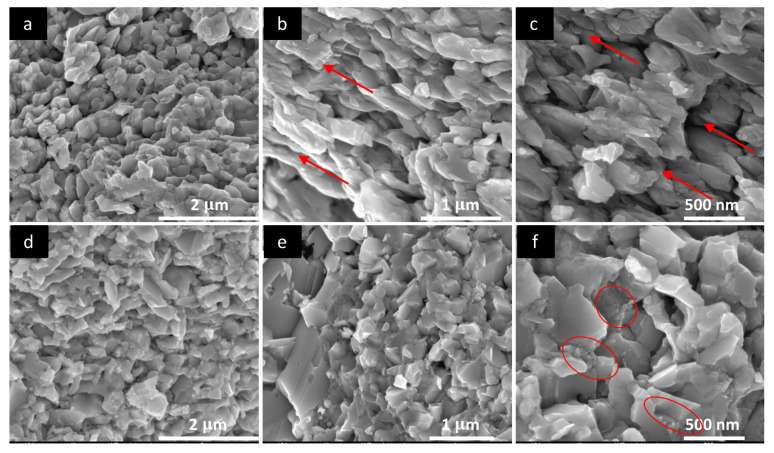
SEM micrographs of SPS sintered Cu_1.8_Se (**a**–**c**) and Cu_2_Se (**d**–**f**) samples at different magnifications. A higher order of similarly sized and partly oriented grains were observed in (**b**) and (**c**) for Cu_1.8_Se (designated by arrows) in comparison with Cu_2_Se (circled regions) in (**d**).

As-made powders were sintered using the SPS technique, which results in densification. SEM micrographs of Cu_1.8_Se and Cu_2_Se after SPS consolidation process are presented in Figure 5. Very fine sub-micron particles are observed in the as-made Cu_1.8_Se in Figure 4a,b. Upon SPS, they form larger grains, on the order of a few hundred nanometers (Figure 5b). The typical grain size ranges from 50 to 500 nm. The large grain size after the SPS process can be explained via increasing mass transfer between grains under high temperature and pressure. In other words, the movement of grain boundary causes the smaller grains to merge inside the larger ones, leading to substantial growth [49]. As-made Cu_2_Se, in Figure 4d,e, possesses larger particles compared to Cu_1.8_Se, which in turn grows to larger grains upon SPS. A careful comparison of Figure 5b,c and Figure 5e,f reveals clear differences in the grain size, compaction, and grain orientation between Cu_2_Se and Cu_1.8_Se samples. Cu_1.8_Se exhibits grains that are more similar in size and more oriented in comparison with Cu_2_Se. Even though there is no strong evidence that explains this issue, there are two possible scenarios. In the first case, this might be due to motion of Cu ions during the SPS process. It is known that Cu_1.8_Se has a higher thermal stability compared to Cu_2_Se, due to copper deficiency [50]. Therefore, Cu ions in Cu_1.8_Se might scatter less and have less random orientation during the SPS process, thus creating more oriented and similar-sized grains in comparison to Cu_2_Se [51].

The second scenario is related to the surface energy of particles. Small particles have higher surface energy and thus show more resistance to combining with other particles to form larger grains under high temperature and pressure, in comparison with larger grains [52]. As seen in Figure 5b,c and Figure 5e,f, Cu_1.8_Se has smaller grains after the SPS process as compared to Cu_2_Se. The difference in grain size is inherited from as-made materials, where higher pressure (see Figure S1a) and temperature during the synthesis of Cu_2_Se resulted in larger particles, as proposed by Hssiang et al. [53]. Grains in Cu_1.8_Se might show more resistance and remain identical during the SPS process, not only due to small (with high surface energy) particles but also due to relatively good thermal stability due to copper deficiency. This will have a strong effect on electron and phonon transport, as indicated by the transport data.

### 3.4. Electronic Transport Properties

**Figure 6:**
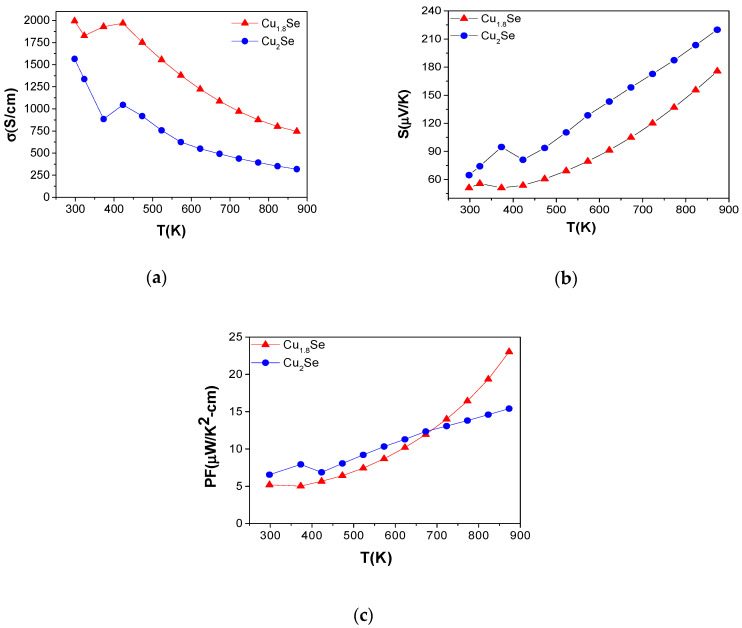
Electronic transport values as a function of temperature for Cu_2_Se and Cu_1.8_Se in the temperature range of 300 to 87 5K: (**a**) electronic conductivity (*σ*), (**b**) Seebeck coefficient (*S*); (**c**) power factor (*S^2^σ*). The non-monotonous behavior of measured quantities in the temperature range of 300–400 K is due to the structural phase transition of Cu_2_Se.

**Figure 7:**
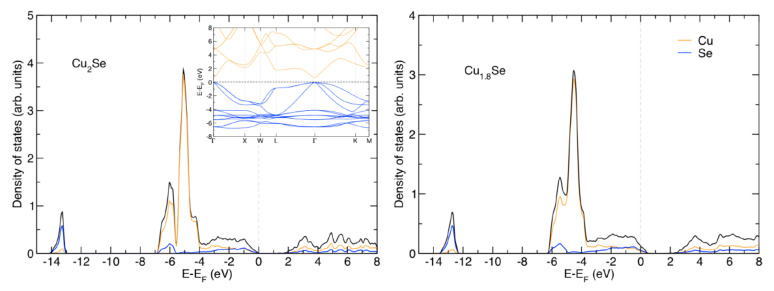
The calculated band structure and density of states (DOS) of Cu_2_Se and the DOS of Cu_1.8_Se. The band structure plot has the valence bands represented by blue color while the conduction bands are given in yellow. For the DOS figures, the total DOS is given by the black line, while the projected DOS for Cu and Se is given in yellow and blue, respectively. The energies are given with respect to the Fermi level, EF.

**Figure 8:**
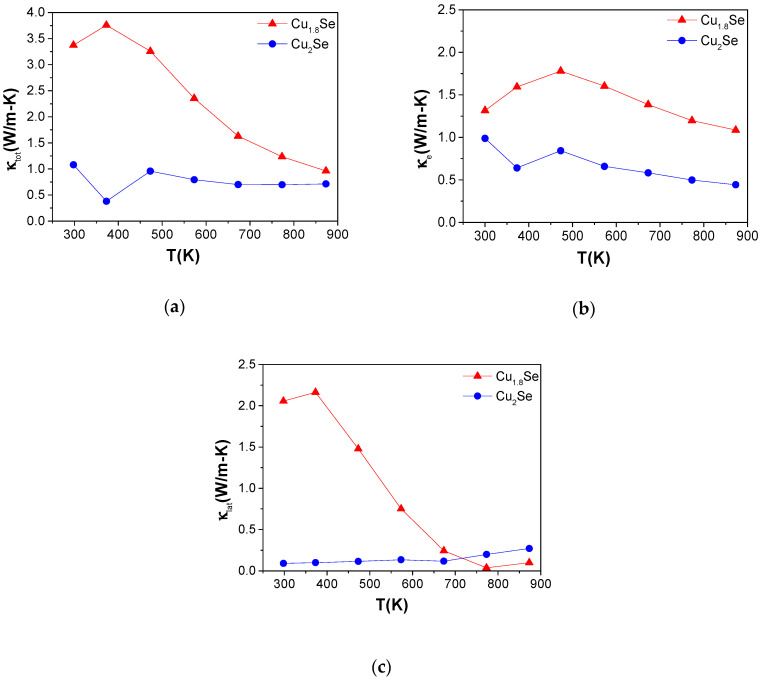
(**a**) Total $\kappa_{tot}$, (**b**) electronic $\kappa_{e}$, and (**c**) lattice $\kappa_{lat}$ thermal conductivity, as a function of temperature for Cu_1.8_Se and Cu_2_Se. The non-monotonous behavior of measured quantities in the temperature range of 300–400 K is due to the structural phase transition of Cu_2_Se.

**Figure 9:**
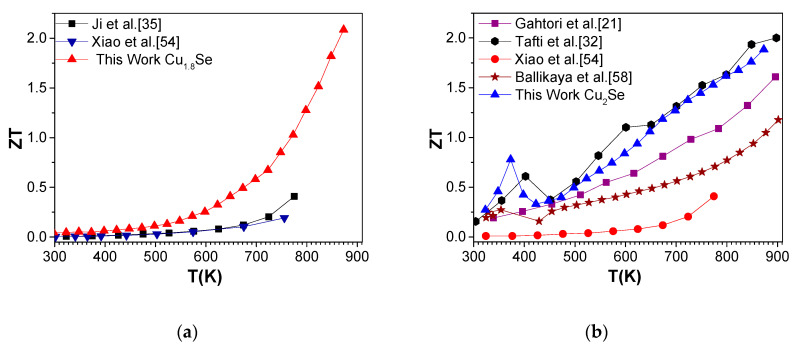
ZT values of (**a**) Cu_1.8_Se and (**b**) Cu_2_Se samples in this work plotted against the earlier data reported for the materials with the same composition. The non-monotonous behavior of measured quantities in the temperature range of 300–400 K in (**b**) is due to the structural phase transition of Cu_2_Se [58].

Transport properties of the SPS sintered pellets have been analyzed in the temperature range of 300–875 K, and the results are presented in Figure 6, Figure 7, Figure 8 and Figure 9. The reported values are average of three measurements at each data point. Seebeck coefficient and electrical conductivity measurements were repeated three times during the heating and cooling cycle in order to observe thermal stability and reproducibility of data. We have seen that there is 1–8% difference of electrical conductivity and Seebeck coefficient values during the heating and cooling process for Cu_2_Se and Cu_1.8_Se (Figures S3 and S4), which is mainly at the low-temperature region. This indicates the relatively good thermal stability of these samples in the temperature range of 300–875 K. However, more than three cycles of measurement are needed, along with additional analyses (such as thermal expansion and pressure test), to obtain actual, long-term thermal stability of these samples. The Cu_2−x_Se is known to be a phase-changing material with a transition temperature in the range 90–110 °C, which is the reason for the sudden dip observed around the transition temperature. The electrical conductivity decreases with temperature, which is a typical highly doped semiconductor behavior (see Figure 6). The highest electrical conductivity is reported for Cu_1.8_Se, followed by Cu_2_Se. This is attributed to the difference in carrier concentration. In other words, Cu_1.8_Se shows higher electrical conductivity because it has higher carrier density due to Cu deficiency [26,54]. The electrical conductivity of Cu_2_Se is consistent with our previous report [11], with slight difference from some of the earlier reports [21,32].

Seebeck coefficient (*S*) versus temperature curves for all the samples are presented in Figure 6b, where the measured positive values reveal a p-type conduction. *S* showed a linear increase with temperature, which also confirms the heavily doped character of these samples. The Cu_2_Se sample exhibited the highest S value, followed by Cu_1.8_Se. Scattering mechanism, carrier density, density of states near the Fermi level, and band structure play important roles in enhancement of *S* [55]. The room temperature Seebeck coefficient value of Cu_2_Se is 60 µV/K and increases to about 220 µV/K, which is roughly 10% greater than values reported by Gahtori et al. [21] (≈ 200 µV/K) and slightly lower than that reported by Yu et al. [56] (≈ 255 µV/K). The trend in magnitude of *S* is inverse that of *σ*, as expected. In other words, samples with high carrier concentration show a small *S* value, as determined by the following equation:

(2) $$S=\frac{8\;\pi^{2}{k_{B}}^{2}T}{3eh^{2}}m^{*}{\left(\frac{\pi }{3n}\right)}^{\frac{2}{3}}\left(1+\gamma \right)$$

where *k_B_*, *T*, *e*, *h*, m*, *n*, and γ are the Boltzmann constant, absolute temperature, electronic charge, Planck’s constant, effective mass of the carrier, carrier concentration, and scattering factor, respectively. Therefore, Cu_1.8_Se shows the lowest ***S*** value, due to its higher carrier density, while the opposite is seen for Cu_2_Se [51,52].

Figure 7 shows the band structure and density of states (DOS) of Cu_2_Se. The electronic properties are similar to what was found by Råsander et al. [45]. Cu_2_Se is found to have a direct band gap at the Γ-point of 0.74 eV between the triply degenerate valence band maximum and the non-degenerate conduction band minimum. Note that in a previous calculation on Cu_2_Se a U = 8.1 eV was required for a gap to open in the system when using LDA+U [45]. With the present PBE+U approach, smaller U-values are required for a gap to open.

The region near the valence band maximum, i.e., between −4 eV and the Fermi level, consists of a mixture of Cu 3d and Se 4p states. Below this region lies a region dominated by Cu 3d states. When Cu vacancies are introduced in the Cu_2_Se lattice, the states are broadened. More importantly, we find a more or less rigid shift of the electronic states with respect to the Fermi level, where the lower number of valence electrons in Cu_1.8_Se (n_e_ = 25.8 per formula unit) compared to Cu_2_Se (n_e_ = 28 per formula unit) shifts the Fermi level into the valence band of a Cu_2_Se-like electronic structure. This makes Cu_2−x_Se systems with x > 0 metallic compared to the semi-conducting electronic structure of Cu_2_Se (Figure 7). Shifting the Fermi level from the valence band maximum into the valence band in an otherwise very similar electron structure will have several effects on the transport properties: (i) the electron conductivity will increase due to the presence of more carriers; (ii) the Seebeck coefficient will decrease due to the metallic character of the states at the Fermi level; and (iii) the thermal conductivity of the electrons will increase. This is also what we find in our measured electronic and thermal conductivities (see Figure 8) as well as for the measured Seebeck coefficient, when comparing Cu_2_Se and Cu_1.8_Se.

The power factors (PF = *S^2^σ*) of these materials have been estimated using the obtained values for *S* and *σ*, and the results are displayed in Figure 6c. PF of samples is estimated to be the highest for Cu_1.8_Se, followed by Cu_2_Se, at 875 Κ. Cu_1.8_Se reached the highest PF (24 µW/K^−2^cm^−1^ at 875 Κ) due to its high electronic conductivity and moderate ***S***. This value is significantly higher than earlier reports that showed 14.2 and 7.5 µW/K^−2^cm^−1^ at 973 K for nanostructured and melt-processed bulk for β-phase, respectively [21]. The PF of the bulk Cu_2_Se (14 µW/K^−2^cm^−1^ is at 875 Κ) is slightly lower than that of earlier reports [56]. There is crossover at 700 K, where PF of Cu_1.8_Se exceeds that of Cu_2_Se. The characteristic behavior of PF factor is dominated by *S* since it is determined by the square of *S*. Therefore, a small a deviation from linearity makes a big difference in the curve. This might be the reason for the observed anomalous increase in PF above 700 K, since the linearity in the *S* of Cu_1.8_Se changes slightly after this temperature, which might be due to increased scattering of charge carriers at this temperature. A high *PF* means a large output power for TE devices, which is an important criterion in addition to *ZT*.

The total thermal conductivities ($\kappa_{tot}$) of the samples are presented in Figure 8. Room-temperature $\kappa_{tot}$ for Cu_1.8_Se showed relatively high values as compared to the rest of the samples. This is likely due to its higher electronic thermal conductivity, $\kappa_{e}\;$. The *κ_tot_* value decreases from 4 to 2 W/m^−1^K^−1^ at 875 K for Cu_1.8_Se, which is a similar to the values reported earlier [21]. In order to detail the assessment of $\kappa_{tot}$, electronic contribution ($\kappa_{e}$) and lattice contribution ($\kappa_{lat}$) of thermal conductivity were calculated (see Figure 8b,c).

The Wiedemann–Franz law can be used to calculate the lattice contribution to the thermal conductivity ($\kappa_{lat}$) of all samples by subtracting the electronic term ($\kappa_{e}=L\sigma T\;$, where *L*, *σ,* and *T* are the Lorenz number, electrical conductivity, and absolute temperature, respectively) from the total thermal conductivity. The *L* values for all compounds were calculated by using the following equation [40]:

(3) $$L={\left[\frac{k_{B}}{e}\right]}^{2}\left[\frac{\left(r+7/2\right)F_{r+5/2}\left(\xi \right)}{\left(r+3/2\right)F_{r+1/2}\left(\xi \right)}-{\left[\frac{\left(r+5/2\right)F_{r+3/2}\left(\xi \right)}{\left(r+3/2\right)F_{r+1/2}\left(\xi \right)}\right]}^{2}\right]$$

where *r* is the charge carrier scattering parameter, *k_B_* is the Boltzmann constant, *e* is the electron charge, and *F_n_*(*ξ*) is the Fermi integral given by:

(4) $$F_{n}\left(\xi \right)=\int_{0}^{\infty }\frac{\chi^{n}}{1+e^{\chi -\xi }}d\chi$$

Here, ξ is the reduced Fermi energy that can be calculated from the Seebeck coefficient *S* and the scattering parameter ***r*** according to:

(5) $$S=\pm \frac{k_{B}\;\left(r+5/2\right)F_{r+3/2}\left(\xi \right)}{e\;\left(r+3/2\right)F_{r+1/2}\left(\xi \right)}-\xi$$

We assumed the system to be highly degenerate and scattering to be dominated by acoustic phonons. Temperature-dependent Lorenz number is estimated as 2.2 to 1.6 × 10^−8^ V^2^/K^2^ in the temperature range of 300–900 K for the analyzed samples. Temperature-dependent $\kappa_{e}$ and $\kappa_{lat}$ values are shown in Figure 8b,c. Both $\kappa_{e}$ and $\kappa_{lat}$ decrease with temperature for all the samples. However, as seen in Figure 8, suppression in the $\kappa_{lat}$ is much more dramatic for the Cu_1.8_Se sample, which can be interpreted by a strong phonon–grain boundary or phonon point defect scattering in this compound. In other words, since Cu_1.8_Se has small grains that are more similar in size and are more oriented, this leads to a high density of grain boundaries. These interfaces in the nanoscale scatter heat carrier phonons (λ _phonons_ ≈ 1 nm) strongly, while electrons (λ _electrons_ ≈ 10–50 nm) are less influenced as they have different energies while travelling in solids [57]. The temperature dependence of $\kappa_{e}\;$ for Cu_1.8_Se confirm this, as there is no significant drop in the $\kappa_{e}$ while there is a significant drop in the $\kappa_{lat}$ at high temperature for this sample.

ZT values are estimated for all investigated samples and are displayed in Figure 9. The general trend is an increase of ZT with increasing temperature for both the samples. Cu_1.8_Se reached an unprecedentedly high ZT value of 2.1 at 875 K, while Cu_2_Se reached 1.9 at the same temperature.

Cu chalcogenide samples with pure phases of Cu_1.8_Se and Cu_2_Se are compared with the values reported in the literature for the corresponding compositions, as displayed in Figure 9. Our pure phase nanostructured Cu_1.8_Se and Cu_2_Se samples (considering this work and our earlier achievement [32]), display a higher overall ZT value in the whole temperature region of investigation. This is mostly due to strongly reduced thermal conductivity values due to nanosized grains behaving as effective phonon scattering centers. Similar results, with a *ZT* value of 2.0, were recently obtained by additional phonon scattering mechanism, via introduction of nano-SiC into Cu_2_Se, as a result of reduced thermal conductivity [59].

## 4. Conclusions

We have demonstrated a highly scalable colloidal synthetic route, using energy-efficient microwave-assisted thermolysis, for the synthesis of nanostructured Cu_1.8_Se and Cu_2_Se. The synthesis process is very rapid and sensitive to the reaction conditions, making it possible to reach different equilibrium phases of Cu_1.8_Se and Cu_2_Se at 200 and 250 °C, respectively. The resulting materials possess very fine particles in the range of 4–30 nm, forming grains on the order of several hundred nm upon sintering. The observed differences between the electronic conductivity characteristics of the two samples have been confirmed by DFT calculations, where we demonstrate the metallic character of Cu-deficient phase Cu_1.8_Se. Due to the synthetic methodology and consolidation process used for the preservation of the nanostructures, an unprecedentedly high ZT value of 2.1 at 875 K was achieved for the nanostructured Cu_1.8_Se, and a high ZT of 1.9 was achieved for the nanostructured Cu_2_Se at the same temperature. The ZT barrier of unity in bulk thermoelectric materials has been easily and reproducibly overcome with these materials. This work demonstrates the promising benefits of the MW-assisted synthesis scheme for highly reproducible and environmentally friendly thermoelectric materials. In combination with carefully controlled SPS process, this leads to high-efficiency thermoelectric materials, thus paving the road for their high-scale production and implementation in niche applications.

## Figures and Tables

**Table 1:** Synthesis and spark plasma sintering (SPS) consolidation parameters for the investigated materials.

Sample	Microwave Syn. Parameters	Heating Rate (°C/min)	SPS Temp (°C)	Load (MPa)	Packing Density (g/cm^3^)	Compaction Density (%)
Cu_1.8_Se	200 °C; 5 min	50	300	75	6.36	95
Cu_2_Se	250 °C; 5 min	50	400	75	6.45	94

**Table 2:** XPS peak fitting results for Cu 2p_3/2_ region for SPS sintered bulk samples.

Samples	BE [eV]	FWHM [eV]	Fraction	Assigned to	Reference
Cu_2_Se	932.3 933.6	1.0 2.5	0.88 0.11	Cu–Se CuO	[47] [48]
Cu_1.8_Se	932.3 934.8	1.0 1.7	0.94 0.06	Cu–Se Cu(OH)_2_	[47] [48]

## Supplementary Materials

The following are available online at https://www.mdpi.com/2079-4991/10/5/854/s1, Figure S1: (**a**) Screenshot of the reactor for experimental design using multivessel rotor; (**b**) multivessel high-pressure rotor used for the MW-assisted synthesis of Cu_1.8_Se and Cu_2_Se nanoparticles. The system can take up to 15 reactors which can be filled up to about 100 mL each, making it possible to synthesize high and reproducible quality nanostructures with a high yield, at a pilot scale, without needing any complicated or reflux systems. A total of 4 positions/vials (out of 15) during the MW-assisted reaction is sufficient to prepare 8–10 g of these materials within a reaction time of 6–8 min. (**c**,**d**) MW synthesis parameters/conditions for Cu_1.8_Se and Cu_2_Se, displaying the temperature and pressure profile during the reaction period. Figure S2: XPS survey spectra for (**a**) Cu_2_Se and (**b**) Cu_1.8_Se. Table S1: Summary of elemental global atomic percentages for bulk Cu_2_Se and Cu_1.8_Se samples. Table S2: XPS peak fitting results for Se 3d region for bulk Cu_2_Se and Cu_1.8_Se samples. Figure S3: Electrical conductivity and Seebeck coefficient measurements of Cu_1.8_Se during the heating and cooling cycle. Figure S4: Electrical conductivity and Seebeck coefficient measurements of Cu_2_Se during the heating and cooling cycle.
