# Supplementary material for: Composition Tuning of Nanostructured Binary Copper Selenides through Rapid Chemical Synthesis and Their Thermoelectric Property Evaluation
Source: Nanomaterials (Basel). 2020 Apr 28;10(5):854. doi: 10.3390/nano10050854 (PMC7712069; doi:10.3390/nano10050854)
Supplement: Supplementary file 1 [file nanomaterials-10-00854-s001.pdf]

Supplementary Material

Composition Tuning of Nanostructured Binary Copper Selenides through Rapid Chemical Synthesis and their Thermoelectric Property Evaluation

Bejan Hamawandi [1], Sedat Ballikaya [2,*], Mikael Råsander [3], Joseph Halim [4], Lorenzo Vinciguerra [1], Johanna Rosen [4], Mats Johnsson [5] and Muhammet Toprak [1,*]

1 Department of Applied Physics, KTH Royal Institute of Technology, SE-106 91, Stockholm, Sweden; bejan@kth.se (B.H.); lorenzo.vinciguerra.25@gmail.com (L.V.)
2 Department of Physics, University of Istanbul, Fatih, Istanbul, 34135, Turkey
3 Applied Physics, Division of Materials Science, Department of Engineering Sciences and Mathematics, Luleå University of Technology, SE-971 87 Luleå, Sweden; mikael.rasander@ltu.se
4 Department of Physics, Chemistry and Biology (IFM), SE-581 83 Linköping, Sweden; johanna.rosen@liu.se
5 Department of Materials and Environmental Chemistry, Stockholm University, SE-106 91 Stockholm, Sweden; mats.johnsson@mmk.su.se
* Correspondence: ballikaya@istanbul.edu.tr (S.B.); toprak@kth.se (M.T.); Tel.: +46-735-519358 (M.T.)

Received: 27 March 2020; Accepted: 26 April 2020; Published: date

MW assisted Synthesis of Cu-chalcogenides

Copper precursor solution was prepared by using a stoichiometric amount of copper acetate placed in to a 20 ml MW vials, with 8 ml oleic acid and 4 ml of octadecene under continuous stirring. The mixture is stirred at 600 rpm for 3 hours. Separately a stoichiometric amount of selenium powder is placed in a 20 ml size MW vial and 2.5 ml of TOP is added with a magnetic stirrer, the mixture stirred at 600 rpm at least for 15 min until all the selenium is completely dissolved. All the synthesis work has been performed using MW-assisted synthesis process, which can be listed among the most energy effective synthesis routes for the production of semiconductor nanoparticles, based on the thermal decomposition of chemical precursors that are otherwise stable at room temperature. One of the biggest advantages of MW-assisted process is the capability of monitoring the temperature and pressure in the reactor. To obtain $Cu_{1.8}Se$, the precursor mixture is kept under stirring at 600 rpm for 2 min and finally inserted into the Microwave reactor (see Fig S1(a) and (b)). The vial has been kept stirred for 5 min before they are heated under MW irradiation to 200 °C, where they were kept for 5 min. (Typical temperature and pressure profiles of the reaction are presented in Fig S1(c) and (d).) The vial is then automatically cooled to room temperature by compressed air. The obtained powders are then washed several times using a 3:1 mixture of Methanol and Hexane. Finally, the powders are placed in a vacuum oven at a temperature of 60 °C and a pressure lower than 10% of atmospheric pressure for 12 hours to ensure total solvent evaporation. This assures batch to batch reproducibility. Typical reaction profiles for synthesis of $Cu_{1.8}Se$ and Cu_2Se are presented in Fig. S1.

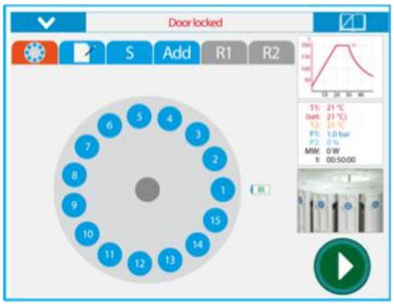

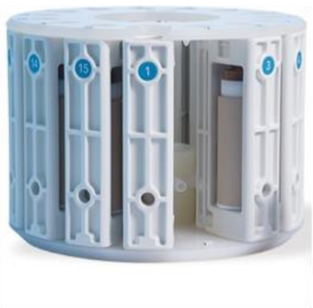

(a) (b)

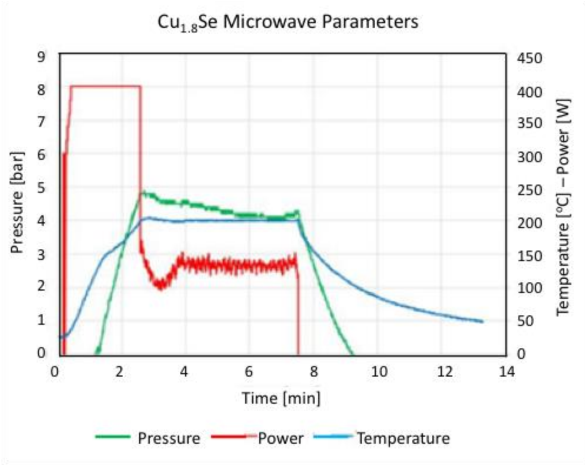

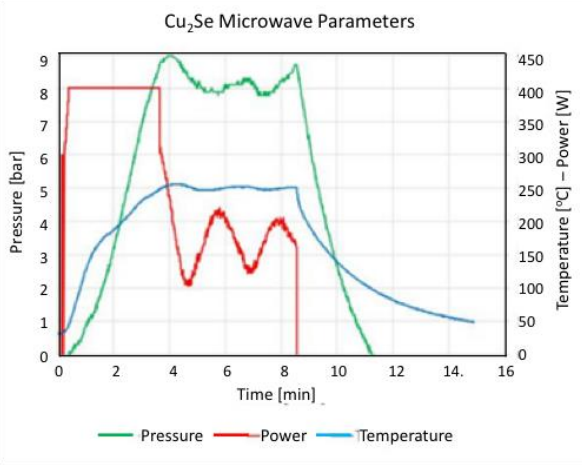

(c) (d)

Figure S1. (**a**) Screenshot of the reactor for experimental design using multivessel rotor; (**b**) Multivessel high pressure rotor used for the MW assisted synthesis of $Cu_{1.8}Se$ and Cu_2Se nanoparticles. The system can take up to 15 reactors which can be filled up to about 100 mL each, making it possible to synthesize high and reproducible quality nanostructures with a high yield, at a pilot scale, without needing any complicated or reflux systems. A total of 4 positions/vials (out of 15) during the MW assisted reaction is sufficient to prepare 8–10 g of these materials within a reaction time of 6–8 min. (**c**,**d**) MW synthesis parameters/conditions for $Cu_{1.8}Se$ and Cu_2Se, displaying the temperature and pressure profile during the reaction period.

To synthesize Cu_2Se the same steps are followed as in the synthesis of $Cu_{1.8}Se$ except one parameter, where the MW reaction temperature is raised to 250 °C.

XPS Analysis of SPS sintered pellets

XPS analyses was performed on SPS sintered pellets in order to establish the composition of compacted pellets for the samples $Cu_{1.8}Se$, and Cu_2Se. The samples were mounted on the sample holder using copper metal strips to hold them in position. The X-ray beam irradiated the sample surface at an angle of 45^0, with respect to the surface and provided an X-ray spot of ≈ 300 x 800 µm. Charge neutralization was performed using a co-axial, low energy (~0.1 eV) electron flood source to avoid shifts in the recorded binding energy (BE). XPS high resolution spectra were recorded for Cu 2p, Se 3d, C 1s, and O 1s. The analyzer pass energy used for all the regions was 20 eV with a step size of 0.1 eV. The BE scale of all XPS spectra was referenced to the Fermi-edge (E_F), which was set to a BE of zero eV. Measurements were done without Ar ion sputtering. The measurements were taken on two different spots on the surface and on the side of each and they showed no difference within the margin of error for each element. The peak fitting was carried out using CasaXPS Version 2.3.16 RP

1.6. Prior to the peak fitting the background contributions were subtracted using a Shirley function. For the Se $3d_{5/2}$ and $3d_{3/2}$ components, the intensity ratios of these peaks were constrained to be 3:2, respectively. The global atomic percentage of the various elements was calculated using the following equation:

$$X_i = 100 \times \frac{A_i}{\sum_{j=1}^{m} A_j} \quad \text{(S1)}$$

where X_i is the atomic concentration of the element i, A_i is the adjusted intensity of element i, and A_j is the total adjusted intensity for all elements. The adjusted intensity is defined as follows:

$$A_i = \frac{I_i}{R_i} \quad \text{(S2)}$$

where I_i is the integrated peak area, and R_i is the relative sensitivity factor.

Summary of elemental global atomic percentages for Cu-Se samples and XPS peak fitting results for Se 3d region for Cu_2Se, and bulk samples are presented in Table S1 and Table S2, respectively.

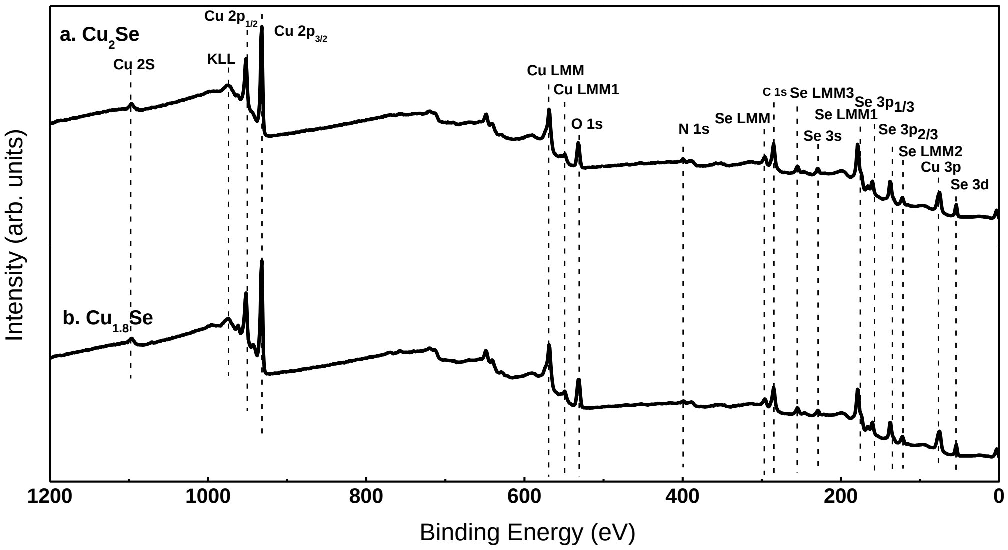

Figure S2. XPS survey spectra for: a. Cu_2Se and b. $Cu_{1.8}Se$.

Table S1. Summary of elemental global atomic percentages for bulk Cu_2Se and $Cu_{1.8}Se$ samples.

Samples	Cu	Se	O	C
Cu_2Se	18.0±0.8	7.8±0.1	18.4±0.5	55.8±0.7
$Cu_{1.8}Se$	18.5±0.4	6.5±0.2	22.8±0.1	52.2±0.3

Table S2. XPS peak fitting results for Se 3d region for bulk Cu_2Se and $Cu_{1.8}Se$ samples.

Samples	BE [eV]	FWHM [eV]	Assigned to	Reference
Cu_2Se	53.8 (54.7) [a]	0.8 (0.7)	Cu-Se	(1)
$Cu_{1.8}Se$	53.9 (54.8) [a]	0.8 (0.7)	Cu-Se	(1)

[a] Values in parenthesis correspond to the $3d_{5/2}$ component. The areal ratios of the Se $3d_{5/2}$ and $2d_{3/2}$ peaks were constrained to 3:2. [Ref 1: .P. Domashevskaya, V. V. Gorbachev, V.A. Terekhov, V.M. Kashkarov, E. V. Panfilova, A. V. Shchukarev, XPS and XES emission investigations of d-p resonance in some copper chalcogenides, J. Electron Spectros. Relat. Phenomena. 114–116 (2001) 901–908.].

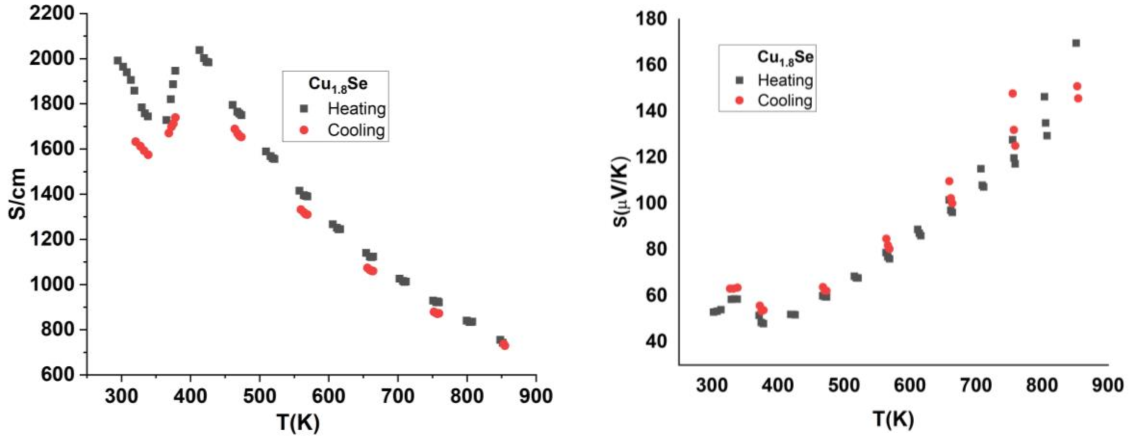

(**a**) (**b**)

Figure S3. Electrical conductivity and Seebeck coefficient measurements of $Cu_{1.8}Se$ during the heating and cooling cycle.

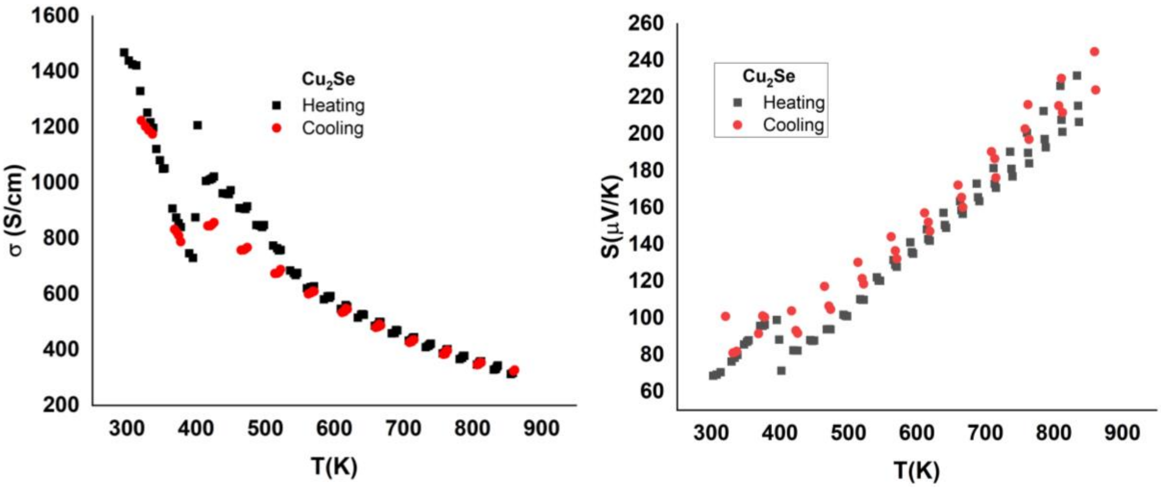

(**a**) (**b**)

Figure S4. Electrical conductivity and Seebeck coefficient measurements of Cu_2Se during the heating and cooling cycle.
